# Dynamics of Transformation from Platinum Icosahedral Nanoparticles to Larger FCC Crystal at Millisecond Time Resolution

**DOI:** 10.1038/s41598-017-16900-6

**Published:** 2017-12-08

**Authors:** Wenpei Gao, Jianbo Wu, Aram Yoon, Ping Lu, Liang Qi, Jianguo Wen, Dean J. Miller, James C. Mabon, William L. Wilson, Hong Yang, Jian-Min Zuo

**Affiliations:** 1 Department of Materials Science and Engineering, University of Illinois at Urbana-Champaign, 1304 W Green St, Urbana, IL 61801 United States; 2 Fredrick Seitz Materials Research Laboratory, University of Illinois at Urbana-Champaign, 104 S Goodwin Ave, Urbana, IL 61801 United States; 3 Department of Chemical and Biomolecular Engineering, University of Illinois at Urbana-Champaign, 600 S Mathews Ave, Urbana, IL 61801 United States; 4 Sandia National Laboratories, Albuquerque, NM 87185 United States; 5 Department of Materials Science and Engineering, University of Michigan, Ann Arbor, MI 48109 United States; 6 Electron Microscopy Center - Center for Nanoscale Materials, Argonne National Laboratory, 9700 South Cass Avenue, Argonne, IL 60439 United States; 7 Present Address: School of Materials Science and Engineering, Shanghai Jiao Tong University, 800 Dongchuan Road, Shanghai, 200240 China

## Abstract

Atomic motion at grain boundaries is essential to microstructure development, growth and stability of catalysts and other nanostructured materials. However, boundary atomic motion is often too fast to observe in a conventional transmission electron microscope (TEM) and too slow for ultrafast electron microscopy. Here, we report on the entire transformation process of strained Pt icosahedral nanoparticles (ICNPs) into larger FCC crystals, captured at 2.5 ms time resolution using a fast electron camera. Results show slow diffusive dislocation motion at nm/s inside ICNPs and fast surface transformation at μm/s. By characterizing nanoparticle strain, we show that the fast transformation is driven by inhomogeneous surface stress. And interaction with pre-existing defects led to the slowdown of the transformation front inside the nanoparticles. Particle coalescence, assisted by oxygen-induced surface migration at *T* ≥ 300 °C, also played a critical role. Thus by studying transformation in the Pt ICNPs at high time and spatial resolution, we obtain critical insights into the transformation mechanisms in strained Pt nanoparticles.

## Introduction

Transformation of metastable structures is a pervasive phenomenon in materials technology^1–4^. The metastable structure, formed at high temperatures, under mechanical stress or by kinetic growth, transforms under the driving force of stored free energy, but the transformation usually requires external mechanical, thermal or chemical triggers. The transformation processes in general are complex that theory can only start to predict^5^. During transformation, atomic motions result in the atomistic structure changes that can be directly observed by X-ray, electron or neutron scattering techniques. Among these, electron scattering based imaging as performed in a TEM provides the highest spatial resolution^2^. However, the time resolution of a conventional TEM is only at a fraction of second (0.033 s at the video rate), compared to nanoseconds in a dynamical TEM^4^. Because of this, boundary motions of durations shorter than a fraction of seconds, but longer than nanoseconds, were not observed by previous studies^6,7^. Their observation presents an opportunity for fast electron detectors^8–10^.

## Experimental Methods

The uniform Pt ICNPs were synthesized using a modified method as described in the methods section. An icosahedral nanoparticle has 6 fivefold axes and 20 triangular faces with the *Ih* symmetry^11,12^. It belongs to a special class of nanostructures called multiply twinned particles (MTP)^13–15^. MTPs have attracted special interests in the fields of cluster physics, quasicrystal, metallic glass, catalysis and nanotechnology because of their noncrystallographic symmetry, and unique chemical and physical properties. The structure of MTPs consists of FCC crystal tetrahedral subunits, which are twin-related on their adjoining faces. The space-filling of those subunits is not complete, leaving a large angular misfit. Consequently, MTPs are believed to be formed with disinclination and related large strain^14–16^. Because of this, large ICNPs have long been predicted theoretically to be unstable compared to the FCC crystal^15,17,18^. Beside the energetic consideration, the particle structure and shape are further influenced by growth kinetics and thermodynamics^19^. Experimentally, as synthesized large ICNPs (or MTPs) are stable in vacuum when observed by a TEM^15,20^. Their stability has been attributed to the extensive atomic rearrangements required to transform to the FCC crystal that is kinetically forbidden under most circumstances^15^.

We investigated the stability of ICNPs under oxidative atmosphere in a newly developed environmental TEM (ETEM). This instrument combines the sample heating and gas environment with a CMOS-based, high-fidelity and high-transfer rate direct electron detection camera (Gatan, Pleasanton, CA, also see^21^), which operates at the imaging speed of 400 frames per second for the time resolution of 2.5 ms in the so-called *in-situ* (IS) mode. An early observation suggested that the morphology of ICNPs was unstable when exposed to oxygen at 210 °C in the original synthetic solution^22^. In this study, the uniform Pt ICNPs were supported on a fine tungsten wire heater^23^, heated to over 300 °C in vacuum and under several gaseous conditions.

## Results and Discussion

**Figure 1:**
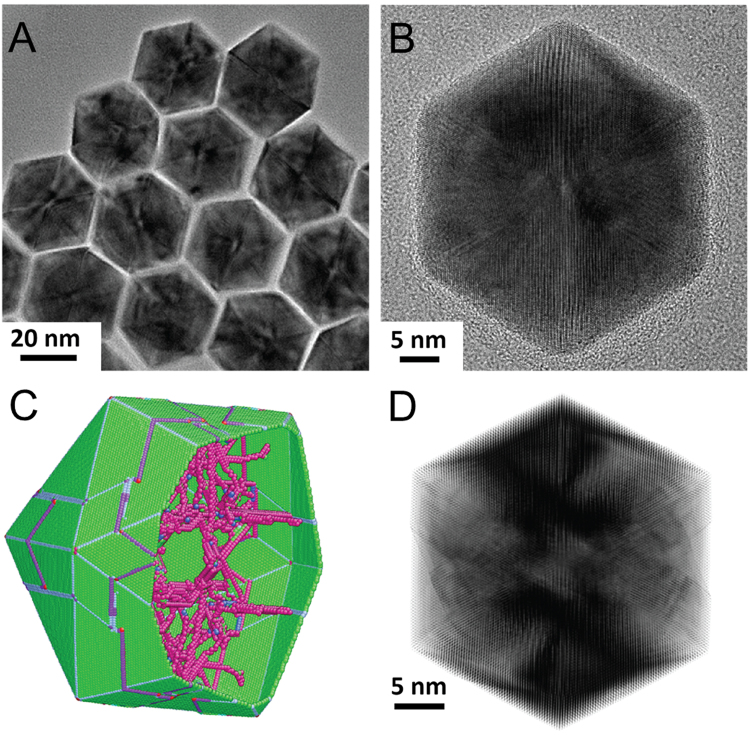
Atomic structure of Pt ICNPs. (**A**) A TEM image of dispersed Pt ICNPs showing the uniform size and structure. (**B**) A high resolution electron micrograph of a Pt ICNP. The ICNP deviates from the 3-fold axis about 5°. (**C**) The atomic model of a Pt ICNP relaxed by MD simulation. In the model, atoms with coordination numbers deviated from that of FCC lattice are colored in purple, showing the positions of defects and dislocations. (**D**) A simulated HREM image using the atomic model in (**C**). The model is tilted 5° about the horizontal axis.

The atomistic structure of as-synthesized Pt ICNPs was investigated first. The as-synthesized nanoparticles were relatively uniform in size and shape, as demonstrated by the TEM image of a group of Pt ICNPs of 36 nm in diameter in Fig. 1A. A nanoparticle with the perfect icosahedral symmetry is expected to have symmetric and homogeneous contrast in the high resolution electron microscopy (HREM) image (for an example of a simulated image, see Fig. S1C in supplementary materials). Using molecular statics simulation (MSS), the relaxed atomic model contains defects, whose locations are demonstrated in Fig. 1C. In Fig. 1D, the simulated HREM image based on the relaxed atomic model shows inhomogeneous contrast similar to that in Fig. 1B. Thus, the inhomogeneous contrast can be attributed to defects in the nanoparticles and related strain. In Figs S2–S3 of supplementary materials, the edge dislocation and the screw dislocation are also resolved in the Pt ICNPs by HREM. Together, the above experimental data demonstrated that as synthesized Pt ICNPs contain defects.

**Figure 2:**
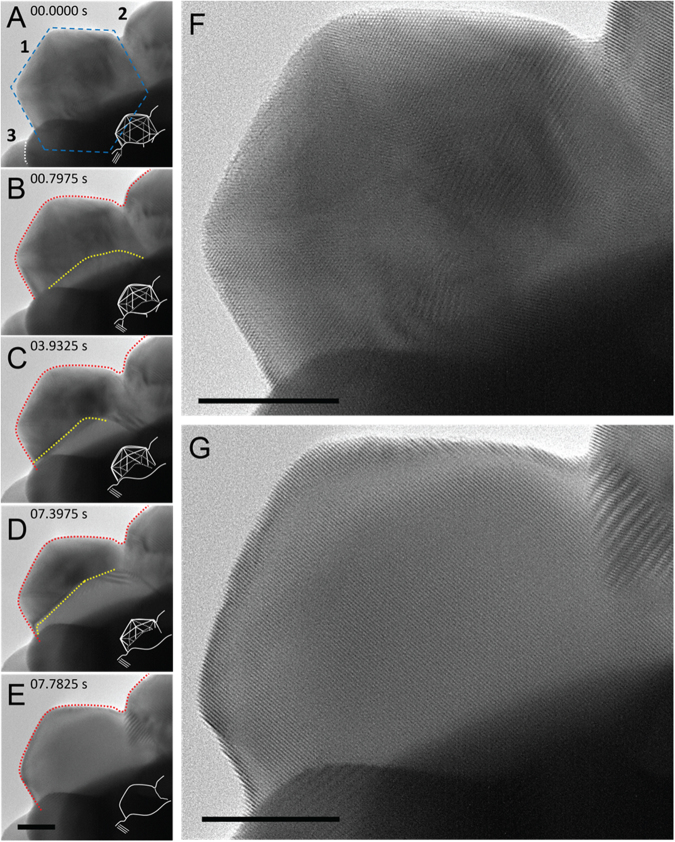
TEM evidence for the transformation of Pt ICNPs to FCC single crystals. (**A**–**E**) Transformation in a Pt ICNP in a time sequence from the start of observation (0 s) to 7.7825 s taken at the electron dose of 90 e/Å^2^s. Figs (**F**,**G**) are the zoom-in images of ICNP 1 before and after transformation (images size 1536 × 1228 pixels). Three Pt ICNPs are seen supported on other nanoparticles as marked in (**A**). The dashed red lines mark the initial surfaces of the ICNPs and boundaries, while the dashed yellow lines mark the transformation front inside ICNP 1 (marked with the blue hexagon in (**A**)), 36 nm in diameter). The insets in (**A**–**E**) at the bottom right corners illustrate the structure and the transformed portion of the particle. The images shown here were averaged over 11 frames recorded at 2.5 ms apart for a displayed time resolution of 27.5 ms. Scale bar, 10 nm.

The transformation from ICNPs to FCC single crystals was observed for clusters of Pt ICNPs that were in contact with each other at 300 °C under the flow of oxygen gas. Figure 2 shows representative TEM images at key stages of the transformation involving three connecting particles (marked as 1, 2 and 3) taken from Supplementary Movie 1. At the start of observation (*t* = 0 s), characteristic contrast was observed from the strained twin boundaries in these particles. The zoom-in HREM image in Fig. 2G shows the atomic lattices in Particle 1 before the transformation. Lattices of different FCC grains were recorded prior to the transformation (Fig. 2F). At 0.7975 s, a bright contrast propagated from the bottom of Particle 1 at the same time as the lattice in Particle 3 rotated (Fig. 2,A,B). Surprisingly, the transformed part of Particle 1 showed the same observed lattice as the new Particle 3. The transformation to a FCC single crystal occurred between 0.7975 and 7.3975 s as the front moved further into Particle 1 and the entire particle transformed into a single crystal at 7.7825 s (Fig. 2E). The single crystal structure was confirmed by the lattice fringes observed in the high resolution TEM micrographs (Fig. 2H) as well as by electron diffraction (see Fig. S4 for an example). The entire transformation of Particle 1 thus took slightly more than 7 seconds once it started.

**Figure 3:**
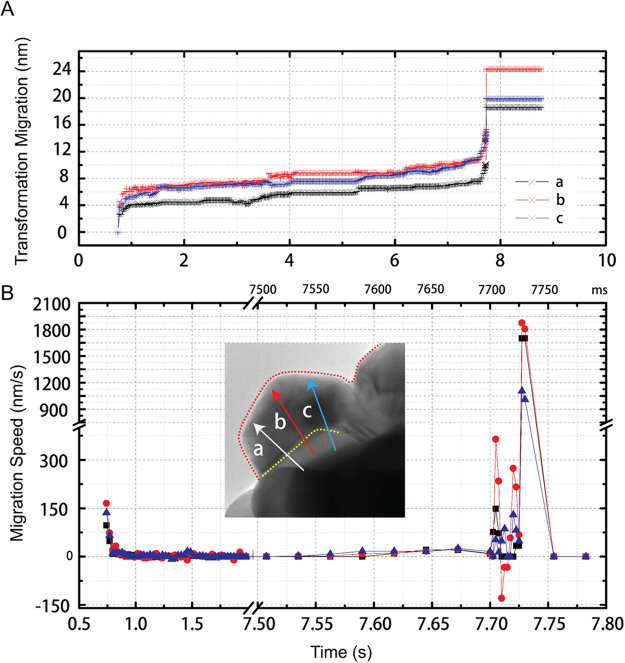
Analysis of boundary movements. (**A**) Transformation and (**B**) migration speed measured along three directions labelled as a, b and c and plotted as a function of time for Pt ICNP 1 shown in Fig. 2. A separate time coordinate is drawn on top of (**B**) at the unit of ms, starting from 7.5000 s.

Figure 3 shows the speed of grain boundary movements in Particle 1. The boundary position and movement were determined by the time-dependent change in contrast recorded in the intensity profiles along three directions of a-c in Fig. 3B. Along all three directions, a fast boundary movement was observed starting at 0.7425 s, followed by a relatively slow and steady propagation from 1 to 7 s. The moving front became unstable after 7.7000 s, as indicated by the back and forth movements (Fig. 3B). The boundary movements ended with an abrupt acceleration at a speed of over 1 μm/s.

**Figure 4:**
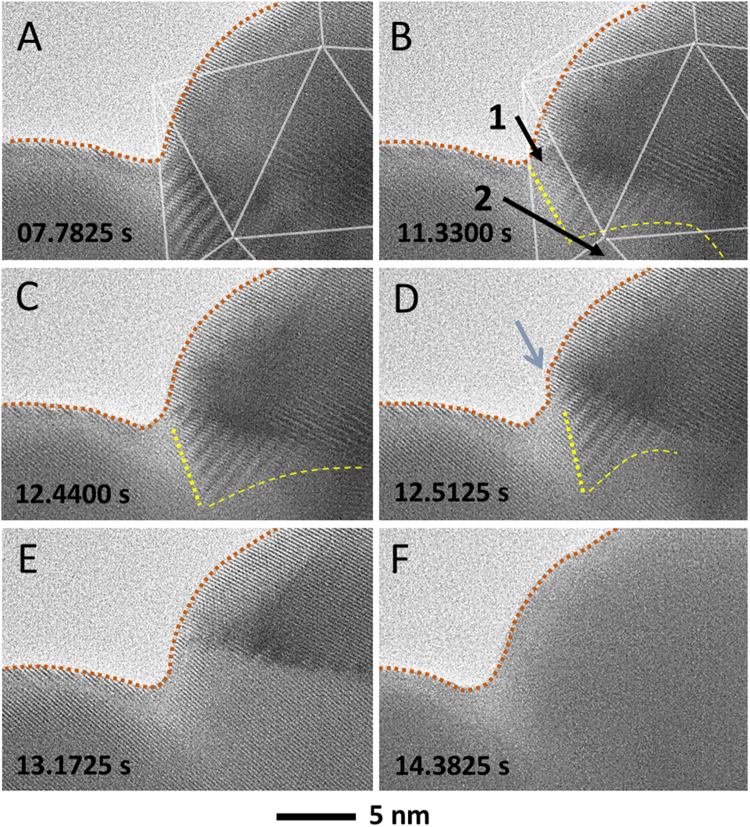
Propagation across the two neighboring Pt particles. (**A–F**) Transformation of Particle 2 (particle on the right) following the transformation of Particle 1 (particle on the left). Particle 2 is superimposed by the partial icosahedral schematic in white. Dotted red lines mark the edge of the particle(s). Dotted yellow lines are used to mark the boundary of Moiré fringe. Dashed yellow lines mark the grain boundaries. The image size are 958 × 658 pixels.

After Particle 1 transformed completely, Particle 2 started to change, with a brief delay, at the region where the two particles were in contact (Fig. 4, Supplementary Movie 2). Moiré fringes were observed first at 7.6175 s due to the overlap of the existing lattice in Particle 2 with the new one in Particle 1 (Supplementary Movie 1). They started to move and disappeared by the time of 14.3825 s, as shown in Fig. 4A–F. The transforming lattice fringes of Particle 2 matched with those in Particle 1, thus the change in the Moiré fringe contrast was the result of transformation fronts propagating in Particle 2. The same process, initially occurring in Particle 1, was also observed for Particle 2, with the slow movement of grain boundary between 11.1925 and 14.3550 s and then the fast one after 14.3550 s (Supplementary Movie 2). The transformation initially started from grains 1 and 2, as marked in Fig. 4B, with one grain in direct contact with Particle 1.

**Figure 5:**
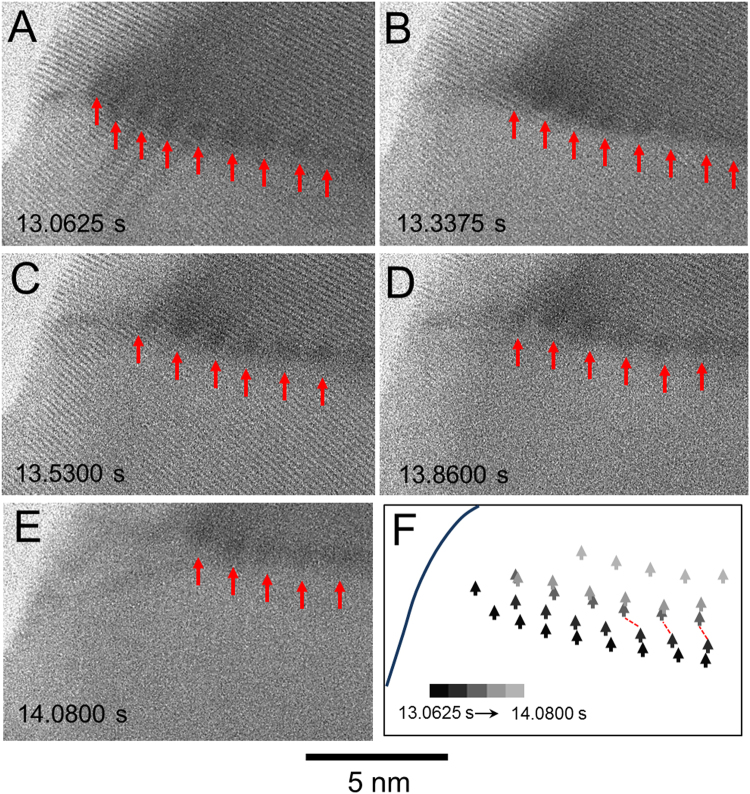
Dislocation motion at the transformation front. (**A**–**E**) TEM images showing the contrast (indicated by red arrows) from dislocations moving with the boundary in Particle 2. (**F**) Illustration of the dislocation track as measured from the series snapshots in (**A**–**E**). The diffusive jump of three dislocations from 13.3375 s (**B**) to 13.5300 s (**C**) is indicated by the red dashed lines in (**F**). The two dislocations on the left most part disappeared as the transformation proceeded.

Dislocation motions accompanied the slow part of the grain boundary movement, which is observed in the middle part of Particle 2, as revealed in a series of TEM images (Fig. 5A–E, Supplementary Movie 2). The movement of dislocation was tracked from 13.0350 to 14.0525 s, as marked by the white triangles in Fig. 5F. These dislocations are associated with the dark blob-like contrast, which comes from the strain close to the dislocations. The nature of these dislocations was confirmed by tracking the {111} lattice fringes on both sides of the grain boundary (Supplementary Fig. S5). The dislocations were observed to move in a concerted fashion, which resulted in the overall forward movement of the transformation front in Particle 2. Two dislocations close to the particle surface as indicated by the red circle disappeared at the early stages of the process, presumably reaching the surface of particle. Individual dislocations also moved sideways, which is consistent with the random nature of dislocation motion by atomic diffusion^24^.

**Figure 6:**
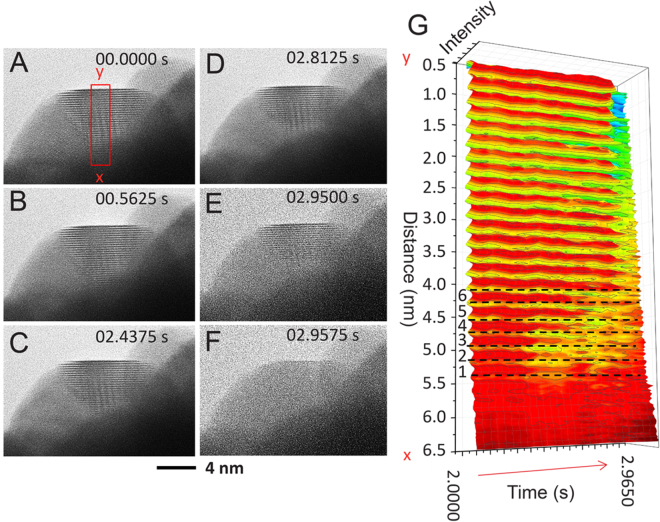
Collective lattice transformation within a single domain of icosahedron. (**A–F**) A series of TEM images show the transformation within a single tetrahedral subunit of an icosahedron along the two-fold axis (image size 870 × 598 pixels). (**G**) The averaged intensity profile along the box from x to y in (**A**) is plotted for each image at different times. Number 1–6 are used to label the first six disappearing lattice fringes.

The last stage of fast transformation was captured for an ICNP oriented along one of the two-fold axes, as shown in Fig. 6A–F (Supplementary Movie 3). Under this imaging condition, one tetrahedral subunit could be recorded with the (111) lattice fringes parallel to the top surface of the particle (Fig. 6A). The lattice fringes were observed initially from the center all the way to the top surface of the Pt particle. During the transformation, the bottom fringes started to fluctuate (Fig. 6B) and disappeared first, as indicated by the intensity profiles in Fig. 6G, taken along the vertical direction between the two positions marked as X and Y in Fig. 6A. These lattice fringes gave rise to oscillations in intensity. We noted that Fringe 1 disappeared first at 2.5000 s, followed by Fringe 2 at 2.8750 s (Fig. 6G). After the disappearance of lattice Fringes 5 and 6, the rest of 16 fringes disappeared simultaneously at 2.9575 s. It took 5 ms for the transformation front to propagate over 3.48 nm, corresponding to a transformation speed of 696 nm/s (Supplementary Fig. S6). This exceptionally fast dynamics occurred in the highly strained regions of the Pt ICNP near the surface, which only became observable using this vastly improved ETEM equipped with the CMOS detector.

## Transformation Mechanism

The Pt ICNPs used in this study possess a very different transformation process from those reported for small MTPs. The small MTPs could co-exist among various metastable states of very small energy differences^25,26^. It has also been suggested that the transformation in small MTPs could enter into and move through a particle via disclination^26^ or continuously involve correlated atomic motion^11^. For the large Pt ICNPs, however, the transformation started from 1 or 2 tetrahedral subunit(s), moved to other ones of different orientations (FCC grains), and eventually formed a larger FCC crystal involving multiple nanoparticles. The abnormal grain growth, which has been observed in a range of polycrystalline materials, is the only other known process that is capable of driving the transformation of a microstructure in a topologically similar way^27–29^. For example, in specially deformed Al containing 0.05% of Si, a few grains grow rapidly during annealing into much larger grains with larger tilt angles than other grains^27^. The transformation of Pt ICNPs is similar to this case in that it does not involve a secondary phase either, except for the support of heating wire. It is also unique because all the grains of Pt ICNPs are of the same shape and size prior to the transformation.

**Figure 7:**
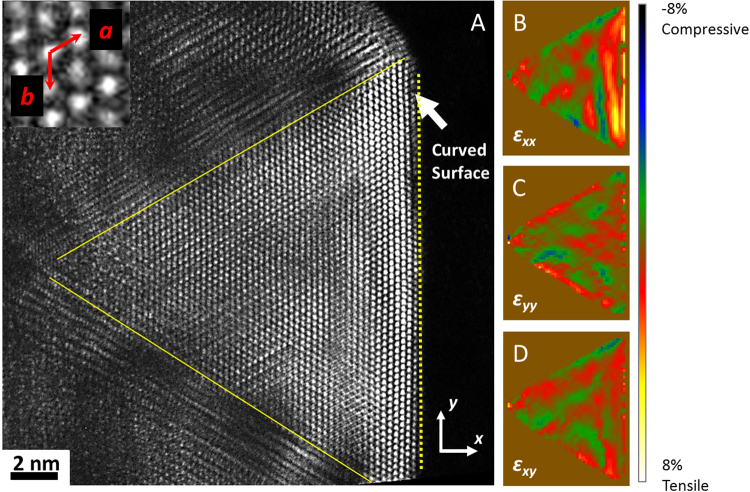
Analysis of strain in a large Pt ICNP: (**A**) dark field aberration corrected TEM image showing the difference in contrast across the nanoparticle, and (**B**–**D**) the corresponding strain maps. (For details, see text).

The major difference between an ICNP and a FCC nanocrystal is the strain. An ideal ICNP is characterized by its disinclinations and related strain^13,15^, however, such ideal model is only applicable to small ICNPs without defects. The question is whether the larger Pt ICNPs can have the Ih morphology but with each segments being unstrained FCC crystals twinned on the {111} planes. To examine this, we performed strain analysis using the atomic resolution images recorded from the Pt ICNPs. In Fig. 7A, the atomic resolution image was recorded in the dark-field mode by selecting a part of the diffraction pattern without the transmitted beam for imaging. To quantify the amount of the projected strain in the image, we measured the strain maps using the TeMA method^30^. In this method, the position of each atomic column was measured first and then used to calculate the strain by comparing the detected atomic positions with a reference lattice. Strain in horizontal and vertical (x and y) directions and the shear strain of the xy plane are shown in Fig. 7B–D. The reference lattice was taken as *a* = *2.74 Å,* *b* = *2.66 Å* and *γ* = *118.70°* as illustrated in the inset of Fig. 7A (the bulk Pt crystal gives *a* = *b* = *2.766 Å* and *γ* = *120°)*. The modulation of strain from the reference deformed lattice can be seen from the strain map. These results together show that the sub unit of the Pt tetrahedron is not the relaxed FCC single crystal, but is strained and the strain is inhomogeneous. Near the surface, which is the right edge on the strain maps of Fig. 7B–D, significant tensile strain was observed in the out-of-plane direction (*ε*_*xx*_). The large strain near the core of the ICNP as predicted by inhomogeneous strain model of Howie and Marks^15^ was not observed in the Pt ICNPs, presumably due to strain relaxation by dislocations as evidenced by the TEM results and the molecular static simulations.

Under our observation conditions, the Pt ICNPs formed aggregates on the heating filament. Prior to the transformation, the ICNPs first coalesced under the oxidative heating environment (see Fig. S7). The oxygen atoms could also diffuse^31^ through the twin boundaries and dislocations in the Pt ICNPs, which modifies the internal stress of the ICNPs. In our experiment, the contact area between ICNPs is seen smoothed during annealing in oxygen (Fig. 4 and Fig. S7C,D). For example, the surface protrusion marked by the arrow in Fig. 4D came from the shear of crystal lattice. It became smooth through surface diffusion under the prolonged heating condition and exposure to oxygen. Therefore, the oxidative annealing promotes the surface diffusion of Pt atoms, which was also seen in ref.^32^.

Experimentally, we observed that isolated ICNPs did not transform under oxidative annealing during the extended observation time (Figs S8–S9), while almost all contacting Pt ICNPs transformed under the same condition with the particle sizes ranging from 15 nm to 36 nm in diameter (Figs S10–13). The difference in the transformation kinetics between the clustered ICNPs and isolated ICNPs suggests a difference in the nucleation of new FCC grain (the nucleation process was not directly observed in our experiment). Since the transformation starts and ends at the particle surfaces, the contact between ICNPs likely provided the initial nucleation grains by the mechanisms of diffusion and coalescence, which was not available to isolated ICNPs. The variation of kinetics at the nanoparticle boundaries likely favored the nucleation of the new FCC grain.

## Conclusion

In summary, clusters of Pt ICNPs are seen to transform into FCC crystals and lose their unique {111} surfaces under the oxidative condition at elevated temperatures. The entire process, which was captured by ETEM at a time resolution of 2.5 ms, includes fast correlative lattice transformation near the nanoparticle surface, and slow grain boundary motion near the nanoparticle center. High resolution electron imaging and molecular statics simulations show inhomogeneous strain and surface stress in the prepared large Pt ICNPs. Further, *in-situ* ETEM observations suggest that interfacial kinetic processes played a critical role in this transformation. Thus, our study demonstrates both fast and slow grain boundary motions in inhomogenously strained Pt ICNPs, and provides key insights into the transformation mechanisms in strained nanoparticles.

## Methods

### Synthesis of Pt ICNPs

The Pt icosahedral nanoparticles were prepared following the previously reported method^22^. Specifically, 20 mg of platinum acetylacetonate (Pt(acac)_2_), 1 mL of dodecylamine (DDA), and 50 μL of oleic acid were mixed and preheated at 130 °C to make the solution **I**. A mixture of 1 mL of diphenyl ether and 9 mL of dodecylamine was degassed in a 25-mL flask under the protection of argon gas for about 10 min. Carbon monoxide gas was then introduced and the CO-saturated solution was heated at 210 °C for 15 min at a flow rate of 120 cm^3^/min (OMEGA FMA-A2305) and a pressure of 10 psi, followed by injecting the solution **I** with a syringe. The mixture was held at 210 °C under CO flow for 30 min. The black precipitate was washed three times with 10 mL of chloroform and collected by centrifugation at 6500 rpm for 8 min.

### *In-situ* ETEM experiments

*In-situ* TEM experiments were conducted in an environmental TEM (Hitachi H9500, 300 kV, base vacuum 10^−5^ Pa). The Pt ICNPs were dispersed in DI H_2_O and then brushed on the tungsten heating wire of the *in-situ* TEM holder, which was heated using an ultrastable DC power supply^23^. The heating temperature was calibrated using the electrical resistance measurement and a thermocouple. A Gatan K2-IS camera was used for recording the ETEM images (image size 1920 × 1856 pixels, electron dose was controlled at 90 e/Å^2^•s). This camera captures images at 400 fps (frame per second) at full frame or up to 1600 fps for 1/4 frame for up to 15 min, recording by a back-thinned CMOS monolithic active pixel sensor^33^. The Pt ICNPs were heated in vacuum, under flowing gas of Ar, N_2_, O_2_ or a mixing gas at a N_2_/O_2_ volumetric ratio (v:v) of 4 and a pressure between 1 × 10^−4^ and 3 × 10^−3^ Pa inside the sample chamber. The gas was introduced using a custom-built gas handling system through a gas injection nozzle inside the sample heating holder. Transformation was observed at T ≥ 300 °C for those ICNPs in contact with each other under the flow of O_2_ or the mixed gas of N_2_ and O_2_ (the latter provided the best imaging results which are shown in this paper).

### High Resolution Electron Microscopy

High resolution electron microscopy (HREM) was carried out using the Argonne chromatic aberration (*C*_*c*_) corrected TEM (ACAT) at the Electron Microscopy Center at Argonne National Laboratory. The TEM is operated at 200 kV. A defocus of 20 nm was employed to image both thick areas in the center of the particle and the thin parts near the edge. Dark field HREM imaging was performed to image the defects in the particles.

### Characterization of Dislocations

To image dislocations in the Pt ICNPs, dark field images were acquired by selecting a sub-area of the diffraction spots excluding the center spot, using the objective aperture. A triangle domain is typically activated as seen in Fig. S7. The inclusion of several spots and the use of aberration correction help to maintain the atomic resolution in the dark field image.

### Image Simulation

HREM images are simulated using the multislice method in Zmult software, with the capability to simulate a large structure consisting up to 2000000 atoms. The parameters used in the simulation was adjusted according to the experimental condition on ACAT (200 keV, *C*_*c*_ = 5 µm, *C*_*s*_ = 1.9 µm, 3.5 mrad convergence angle). A defocus of 20 nm was selected to image both thick part in the particle center and the thin part close edges. The Pt ICNP atomic models were slightly tilted to match with experimental images.

### Molecular Statics Simulation

Molecular statics simulations were carried out in LAMMPS with embedded atom model (EAM) interatomic potential of Pt^34^. Pt ICNP with different diameters of 5, 15 and 30 nm, respectively, were built based on previous literature^35^. Conjugate gradient (CG) minimization was applied to relax the atomistic structures of the whole nanoparticles. There were only twin boundaries but no pre-existing dislocations inside these nanoparticles before relaxations. After the relaxations, a certain amount of dislocations were automatically generated only in the nanoparticle with the diameter of 30 nm as shown in Fig. 1(C), but no dislocations were observed in those smaller nanoparticles. This is because that the total strain energy inside the nanoparticle should be larger than the critical value for dislocation nucleation in this nanoparticle and it is proportional to the nanoparticle volume.

## Electronic supplementary material

Supplemental Figures

Suppl. movie 1

Suppl. movie 2

Suppl. movie 3
