# Supplementary material for: Dynamics of Transformation from Platinum Icosahedral Nanoparticles to Larger FCC Crystal at Millisecond Time Resolution
Source: Sci Rep. 2017 Dec 8;7:17243. doi: 10.1038/s41598-017-16900-6 (PMC5722898; doi:10.1038/s41598-017-16900-6)
Supplement: Supplementary file 1 — Supplemental Figures [file 41598_2017_16900_MOESM1_ESM.doc]

Supporting Information

**Dynamics of Transformation from Platinum Icosahedral Nanoparticles to Larger FCC Crystal at Millisecond Time Resolution**

Wenpei Gao^1,2, ‡^, Jianbo Wu^1,2,3, †,‡^, Aram Yoon^1,2^, Ping Lu^4^, Liang Qi^5^, Jianguo Wen^6^, Dean J. Miller^6^, James C. Mabon^2^, William L. Wilson^1,2^, Hong Yang^3^,*, Jian-Min Zuo^1,2^,*

^1^Department of Materials Science and Engineering, University of Illinois at Urbana-Champaign, 1304 W Green St, Urbana, IL 61801, United States
^2^Fredrick Seitz Materials Research Laboratory, University of Illinois at Urbana-Champaign, 104 S Goodwin Ave, Urbana, IL 61801, United States ^3^Department of Chemical and Biomolecular Engineering, University of Illinois at Urbana-Champaign, 600 S Mathews Ave, Urbana, IL 61801, United States ^4^Sandia National Laboratories, Albuquerque, NM 87185, United States ^5^Department of Materials Science and Engineering, University of Michigan, Ann Arbor, MI 48109, United States ^6^Electron Microscopy Center - Center for Nanoscale Materials, Argonne National Laboratory, 9700 South Cass Avenue, Argonne, IL 60439, United States

Present Addresses

† Current address: School of Materials Science and Engineering, Shanghai Jiao Tong University, 800 Dongchuan Road, Shanghai 200240, China.

AUTHOR INFORMATION

Corresponding Author

*Corresponding Author: [jianzuo@illinois.edu](mailto:jianzuo@illinois.edu), [hy66@illinois.edu](mailto:hy66@illinois.edu)

Figs. S1 to Fig. S13 and 3 movies.

**Supplementary Movies**

**Movie. S1.** The rapid transformation of in Pt ICNP 1 to FCC single crystal shown in Fig. 1. The movie was captured at 400 fps and played at real time speed.

**Movie. S2.** The rapid transformation from ICNP 1 to ICNP 2 shown in Fig. 4. The movie was captured at 400 fps and played at real time speed.

**Movie. S3.** Collective lattice transformation within a single domain of icosahedron shown in Fig. 5. The movie was captured at 400 fps and played at real time speed.


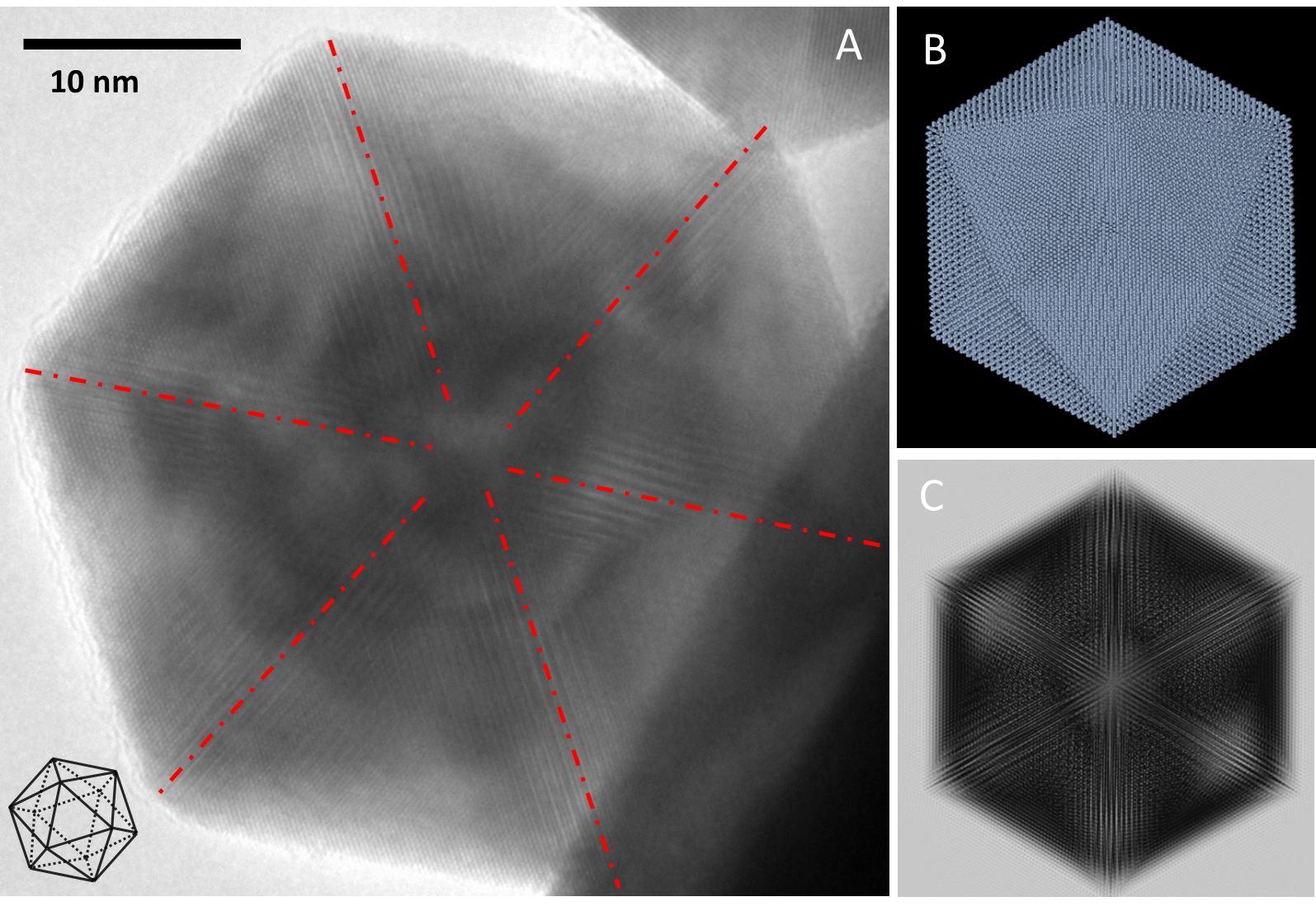
**Supporting Figures (S1 to S13)**

**Fig. S1.** **Structure of Pt ICNPs.** (**A**) Representative HRTEM image recorded along the 3-fold axis of the ICNP. A 3D schematic of the icosahedral particle structure is shown in the inset. Along the 3-fold symmetry axis, the 2D TEM image of the Pt ICNP appears hexagonal. Six projected triangle domains can be seen simultaneously with their joining twin boundaries and lattice fringes along this projection. The projected triangle domains are shown by the red lines. (**B**) A Pt ICNP model based on Mackay icosahedron. (**C**) Simulated HREM image using the atomic model shown in (**B**), shows the symmetric contrast with characteristic features along the multiple twin boundaries.

**
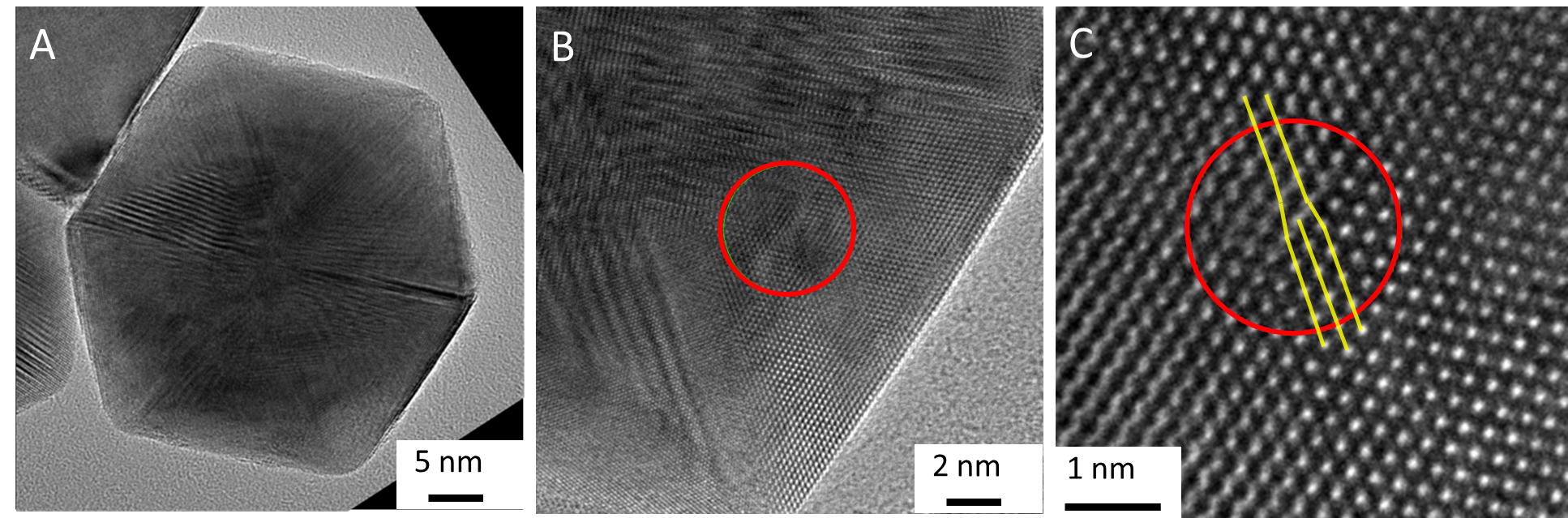
**

**Fig. S2. Edge dislocation in large Pt ICNPs.** Representative HR-TEM images of (**A**) a Pt ICNP, (**B**) its bottom right tetrahedral region and (**C**) an edge dislocation marked by the yellow lines. Heterogeneity and edge dislocations within the Pt ICNP can be clearly visualized.

**
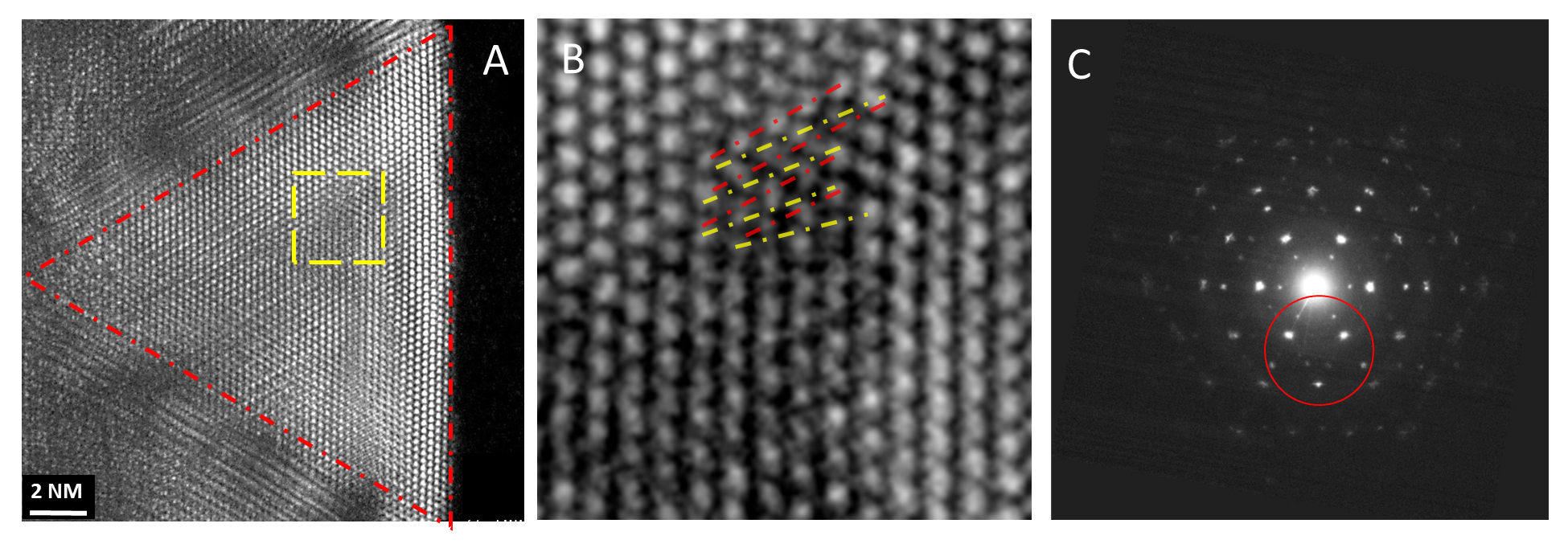
**

**Fig. S3. Screw dislocation in Pt ICNPs.** (**A**) Dark field HR-TEM image of a domain within a Pt ICNP, (**B**) the atomic structure and (**C**) nano beam diffraction (NBD) pattern of the boxed region. Screw dislocation, which is indicated by the red and yellow dash lines in Panel B, can be readily identified in the Pt ICNP.

**
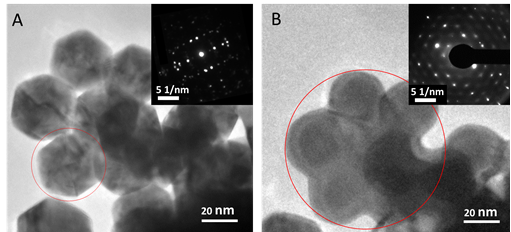
**

**Fig. S4.** **Environmental TEM images and their corresponding nano beam diffraction patterns (insets) of Pt ICNPs (A) before and (B) after the transformation into an fcc phase single crystal.** The NBD from a single Pt ICNP was taken on the particle in the red circle in (A), showing a diffraction pattern with icosahedral symmetry. The particles transformed upon annealing in the mixed gas of O_2_:N_2_ at 1:4 (v:v) at 300 ºC. After transformation, the entire Pt ICNP cluster has transformed into FCC structure, as evidenced by the diffraction pattern in the insets of (B), taken on the area with in the red circle. The transformation took places at all areas, where Pt ICNPs are in contact.

**Fig. S5.** **TEM micrograph showing the dislocation associated with the dark blob-like contrast.** In this zoomed-in image of the grain boundary in NP 2. The {111} lattice fringes on both sides of the grain boundary are highlighted in red in several areas. Edge dislocations can be determined by the lattice mismatch. These dislocations are associated with the dark blob-like contrast observed in Fig. 4 of the main text. The dark contrast arises from the strain close to the dislocations. In the image series, the lattice fringes are not always seen in each image, while the dark contrast persists in every frame throughout the experiment as shown in the videos. The dark contrast was used to help locate the dislocations, and keep track of their movements.


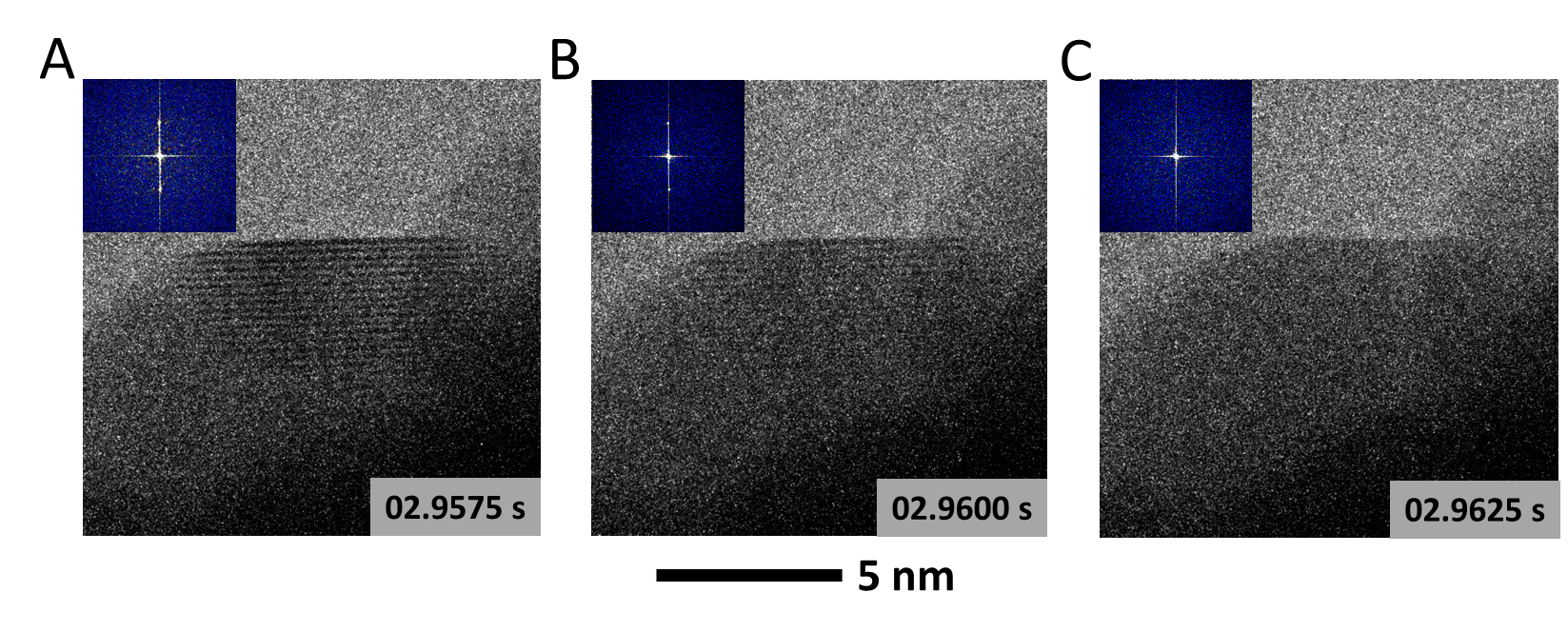


**Fig. S6.** **TEM images showing the collective lattice transformation.** The TEM images show the last part of grain boundary migration towards the surface of a single domain in a 2D projection of a Pt icosahedron. The grain boundary migrated within a single domain in a 2D projection of a Pt icosahedron at (**A**) 02.9575 s, (**B**) 02.9600 s, and (**C**) 02.9625 s, respectively. With the time lapse of 2.5 ms between each image, at 02.9575 s the lattice fringes were seen and evidenced by the FFT spectrum in the inset. At 02.9600 s the contrast of lattice fringes became dimmer, indicating the lattice transformation. The existence of the remaining lattice was evidenced by the FFT spots in the inset at the reduced intensity. At 02.9625 s the lattice fringes disappeared, as evidenced by the FFT in the inset, representing the completion of the transformation.


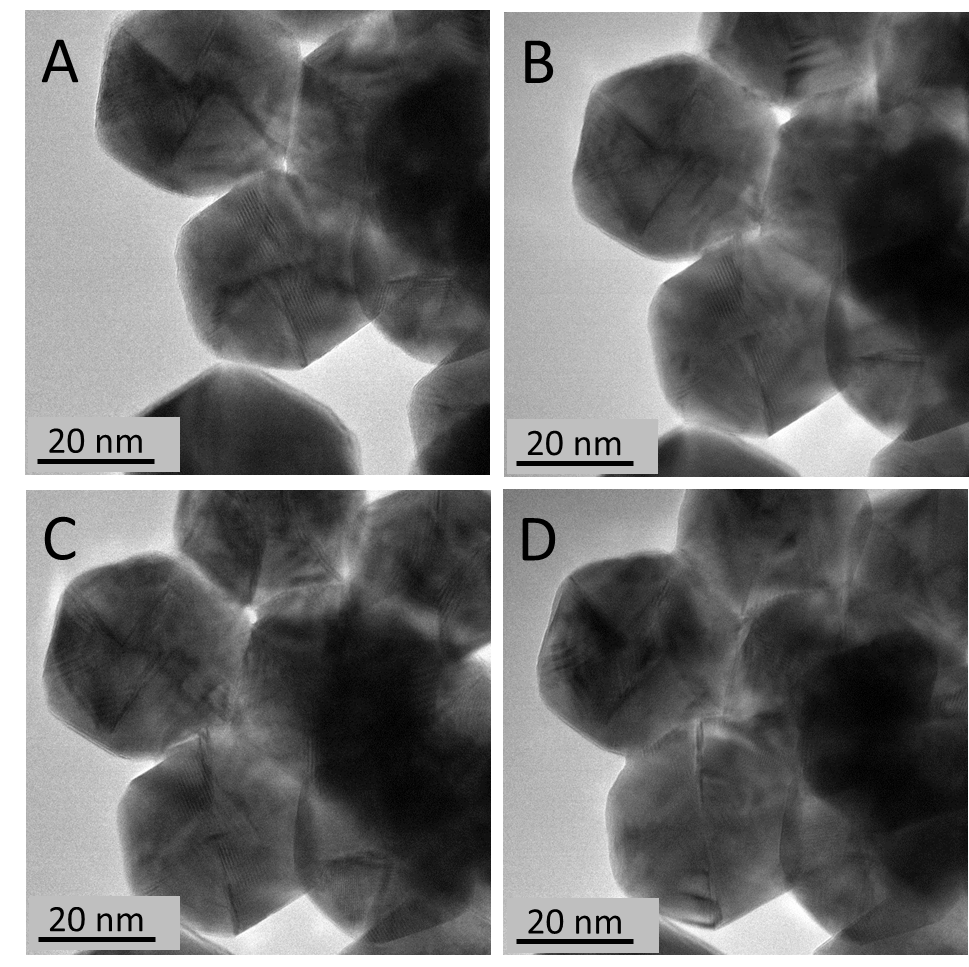


**Fig. S7.** **Environmental TEM images of clusters of Pt ICNPs.** Images of the same group of Pt ICNPs in Fig. S4 tracked (**A**) before and (**B**) after in-situ heating treatment in vacuum at 300 ºC for 30 min, and (**C**) and (**D**) before and after heating in the mixing gas of O_2_:N_2_ at 1:4 (v:v) at 200 ºC for 10 min, respectively. No obvious changes except for some corner rounding were observed after heating in vacuum at 300 ºC for 30 min, the contrast from the icosahedral structure and multiply twinned boundaries remained visible in (B). However, heating in oxygen at 200 ºC results in the agglomeration of particles and disappearance of gaps between the particles in (D).


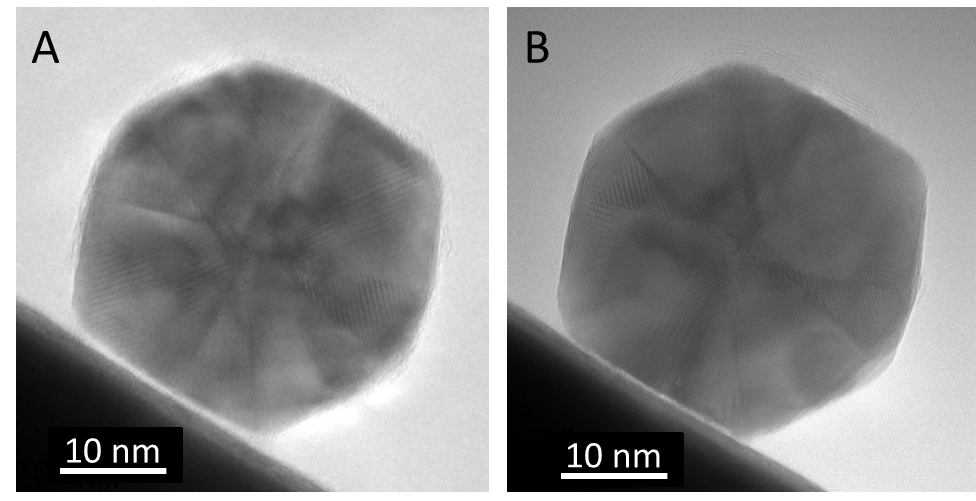


**Fig. S8.** **Isolated individual Pt ICNPs in in-situ oxidative annealing experiment.** TEM images of representative Pt ICNP (A) before and (B) after exposing to the mixing gas of N_2_ and O_2_ at 4:1 (v:v) ratio. The treatment was conducted at 300 ºC for 10 min. No obvious change was observed.


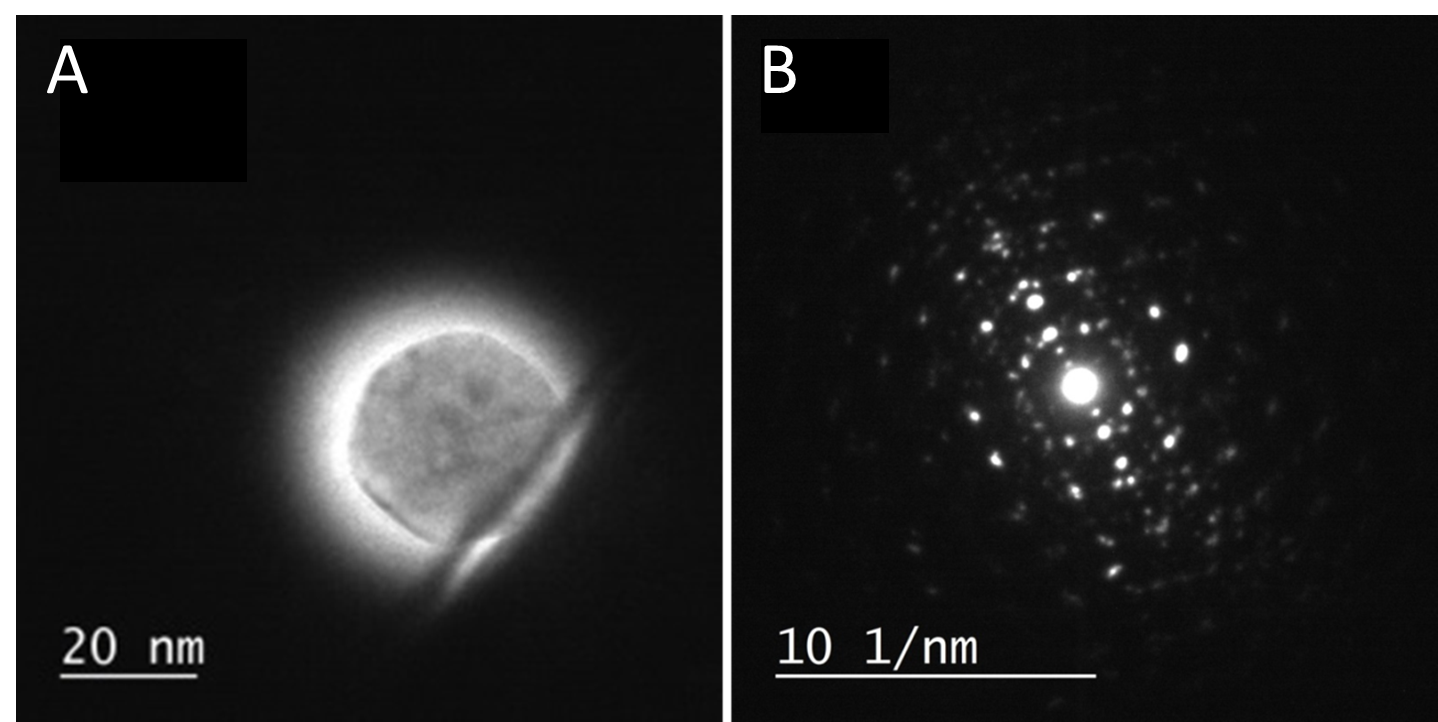


**Fig. S9.** **Nano beam diffraction of isolated individual Pt ICNPs after oxidative annealing experiment.** (A) TEM image and (B) the nano beam electron diffraction pattern of a selected Pt ICNP after oxidative annealing. This Pt ICNP was pretreated in the mixing gas of N_2_ and O_2_ at 4:1 (v:v) ratio at 400 ºC for 30 min. Our result shows the icosahedral symmetry remains intact.

**
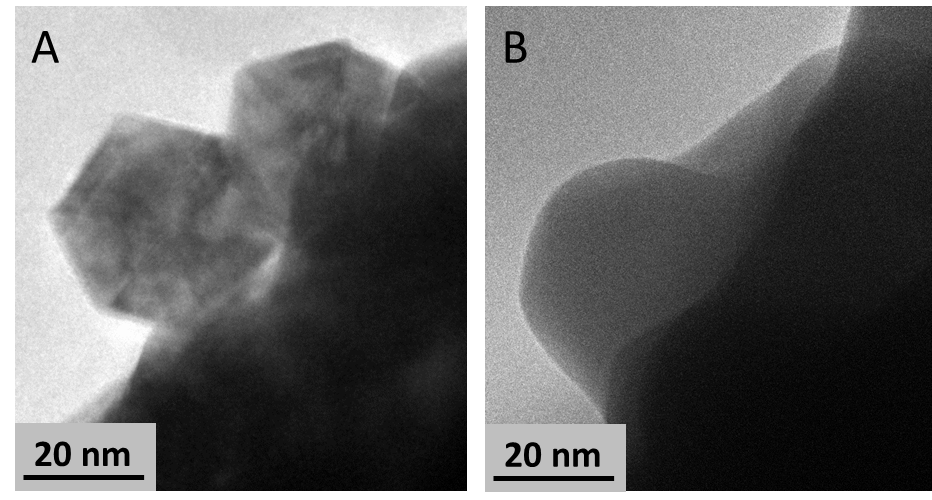
**

**Fig. S10.** **TEM images showing two 36-nm ICNPs on a group of nanoparticles before (A) and after (B) the transformation in the mixing gas of O_2_:N_2_ at 1:4 (v:v) at 300 ºC.**

**
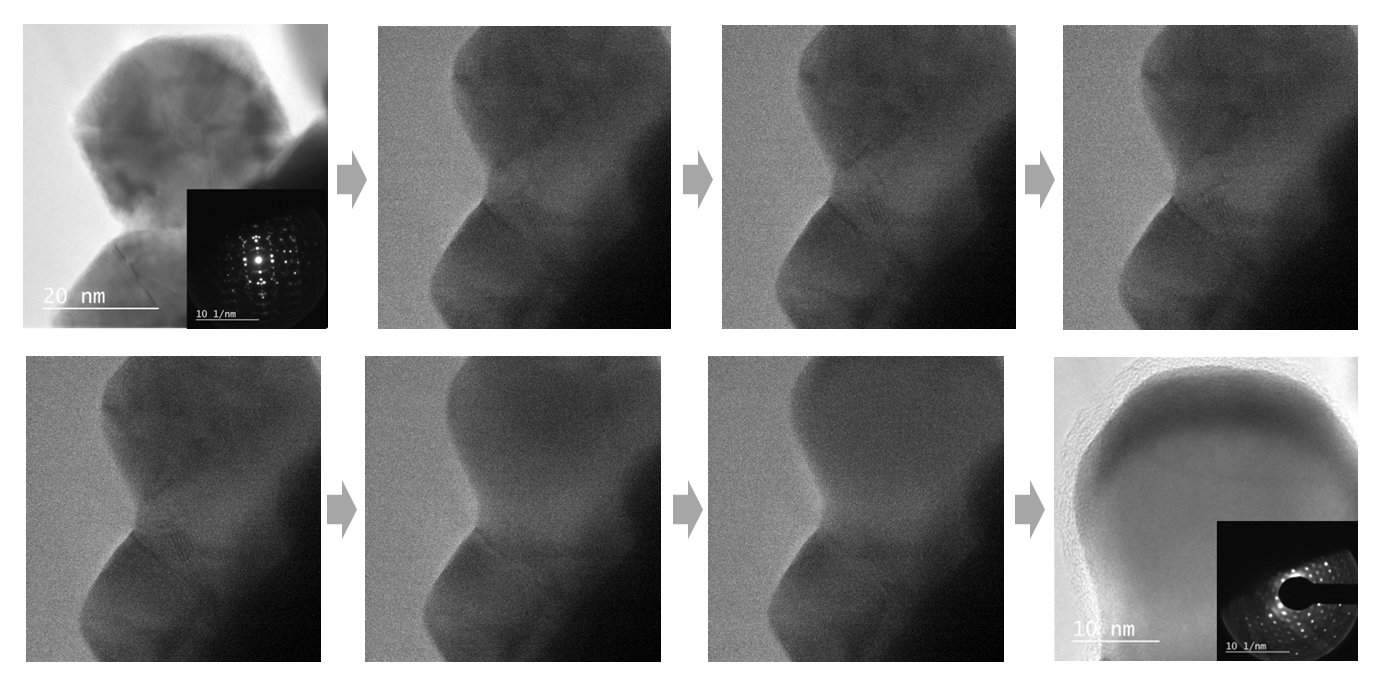
**

**Fig. S11.** **A series of TEM images showing the transformation of 36-nm ICNPs.** An intact Pt ICNP in contact with other ICNPs is shown in the first image, with the inset showing the nano beam diffraction pattern. The particles transformed upon annealing in the mixed gas of O_2_:N_2_ at 1:4 (v:v) at 300 ºC. The resulting particle is shown in the last image, where the facets of ICNPs were lost The inset shows a nano beam diffraction pattern from an fcc structure.

**
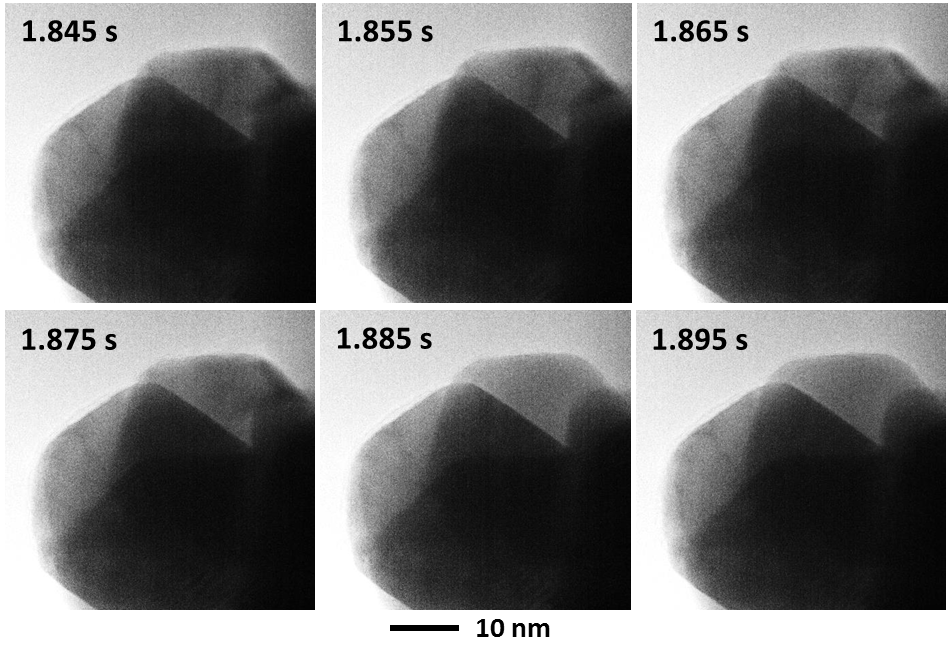
**

**Fig. S12.** **TEM images showing the transformation of a different ICNP.** The top half of this ICNP transformed completely between 1.875 s and 1.885 s.

**
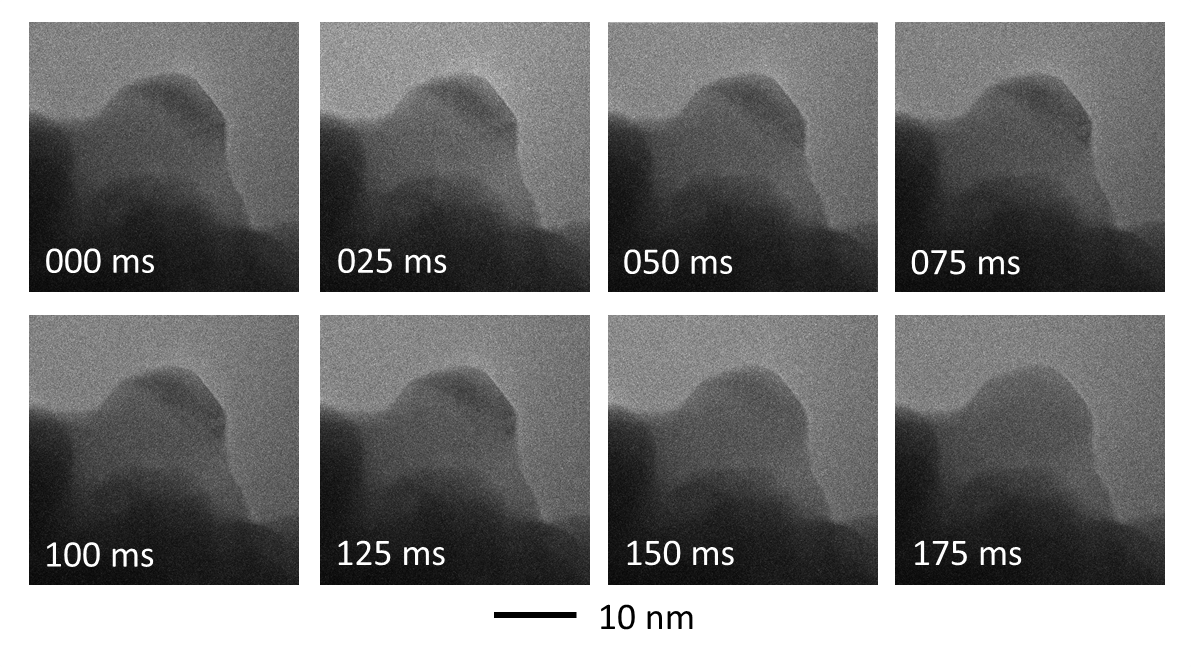
**

**Fig. S13.** **TEM images showing the transformation of 15-nm ICNPs.** Half of the top ICNP transformed into single crystal within 175 ms.
